# Determinants of Functional Coupling between Astrocytes and Respiratory Neurons in the Pre-Bötzinger Complex

**DOI:** 10.1371/journal.pone.0026309

**Published:** 2011-10-19

**Authors:** Christian Schnell, Jens Fresemann, Swen Hülsmann

**Affiliations:** 1 Abt. Neuro- und Sinnesphysiologie, Zentrum Physiologie und Pathophysiologie, Georg-August-Universität, Göttingen, Germany; 2 DFG-Center Molecular Physiology of the Brain (CMPB), Göttingen, Germany; University of Muenster, Germany

## Abstract

Respiratory neuronal network activity is thought to require efficient functioning of astrocytes. Here, we analyzed neuron-astrocyte communication in the pre-Bötzinger Complex (preBötC) of rhythmic slice preparations from neonatal mice. In astrocytes that exhibited rhythmic potassium fluxes and glutamate transporter currents, we did not find a translation of respiratory neuronal activity into phase-locked astroglial calcium signals. In up to 20% of astrocytes, 2-photon calcium imaging revealed spontaneous calcium fluctuations, although with no correlation to neuronal activity. Calcium signals could be elicited in preBötC astrocytes by metabotropic glutamate receptor activation or after inhibition of glial glutamate uptake. In the latter case, astrocyte calcium elevation preceded a surge of respiratory neuron discharge activity followed by network failure. We conclude that astrocytes do not exhibit respiratory-rhythmic calcium fluctuations when they are able to prevent synaptic glutamate accumulation. Calcium signaling is, however, observed when glutamate transport processes in astrocytes are suppressed or neuronal discharge activity is excessive.

## Introduction

Breathing is a multifaceted behavior that is dependent on activity of neuron populations in the medulla oblongata and the pons and modulated by supra-bulbar and spinal neural networks [1], [2], [3]. An important functional feature of neurons in this network, including the pre-Bötzinger Complex (preBötC), is the occurrence of rhythmic bursts of action potentials, which are accompanied by parallel increases of potassium ions in the extracellular space [4], [5] and release of neurotransmitters and neuromodulators [6], [7], [8]. Astrocytes maintain homeostasis of the extracellular space by regulating the extracellular concentration of neurotransmitters such as glutamate [9], [10] or glycine [11]. Major disturbances of astrocyte transmitter uptake can impair respiratory activity e.g. by interfering with the glutamine-glutamate cycle and with synaptic transmission [6], [11], [12], [13]. Astrocytes express K^+^ channels (Kir4.1; KCNJ10) that maintain potassium homeostasis and the resting membrane potential of astrocytes in the medulla [14]. Several authors recently reported that astrocytes in the respiratory network respond to prevailing neuromodulators with an increase of intracellular calcium concentration [15], [16], [17]. Two consequences of elevated [Ca^2+^] in astrocytes have been suggested: astroglial neurotransmitter release that influences activity of nearby neurons [18], [19], [20], [21], and effects on central CO_2_/pH-chemosensitivity [17].

In the present study, we tested whether astrocytes exhibit membrane properties or calcium signals that correlate with ongoing activity of neighboring respiratory neurons. We obtained whole-cell recordings from fluorescently labeled astrocytes and performed 2-photon calcium imaging experiments on rhythmic slice preparations to determine the degree of functional coupling between astrocytes and neurons in the preBötC.

## Results

### Rhythmic currents can be measured in astrocytes of the pre-Bötzinger complex

**Figure 1:**
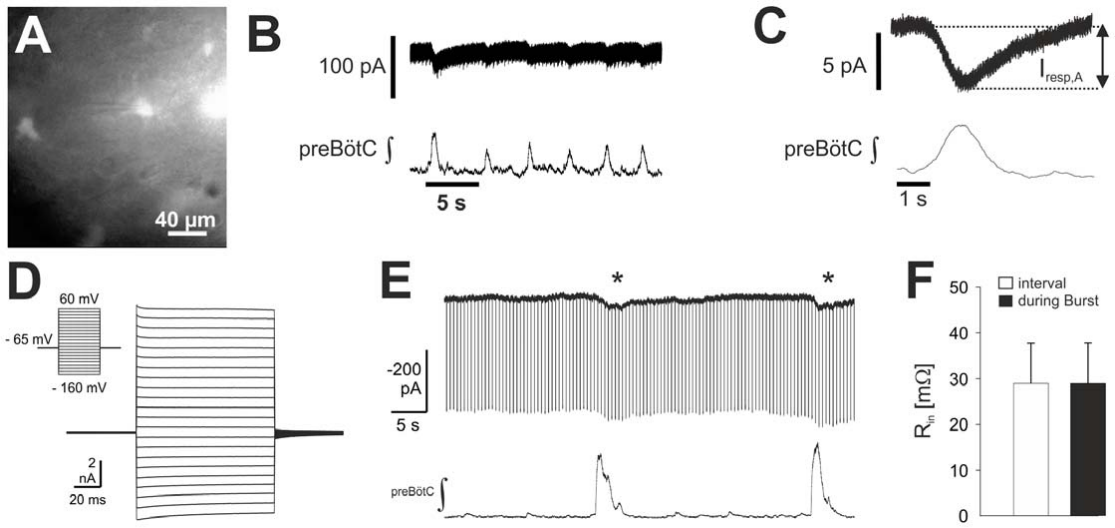
Rhythmic inward currents in astrocytes of the pre-Bötzinger Complex (preBötC). (**A**) To identify astrocytes a CCD-image was taken and the astrocyte, identified by its (green) fluorescence in the center of the image was whole-cell recorded in voltage-clamp mode showing (**B**) respiratory-rhythmic inward currents that were partly obscured by the noise (V_hold_ = -70 mV; upper trace). The integrated preBötC-field potential (preBötC ∫), recorded in parallel, is shown in the lower trace. **(C)** Cycle triggered averaging of inward currents was performed, using preBötC-field potentials as triggers to allow the measurement of the amplitude of the respiratory rhythmic current (I_resp,A_). (**D–F**) Input resistance of the astrocytes remains unchanged during astrocytic inward currents: Panels (**D**) and (**E**) show whole-cell recordings taken from a fluorescent preBötC astrocyte. (**D**) Current traces recorded in response to the voltage step protocol, show in the insert, identified this astrocyte as passive. (**E**) In the presence of bicuculline (20 µM), large amplitude preBötC field potentials were accompanied by large inward currents (asterisks) in the astrocytes (D). Hyperpolarizing voltage steps (−10 mV) were applied to the astrocyte to measure membrane input resistance (R_in_), which did not change in association with inward current transients (**F**; n = 3).

**Figure 2:**
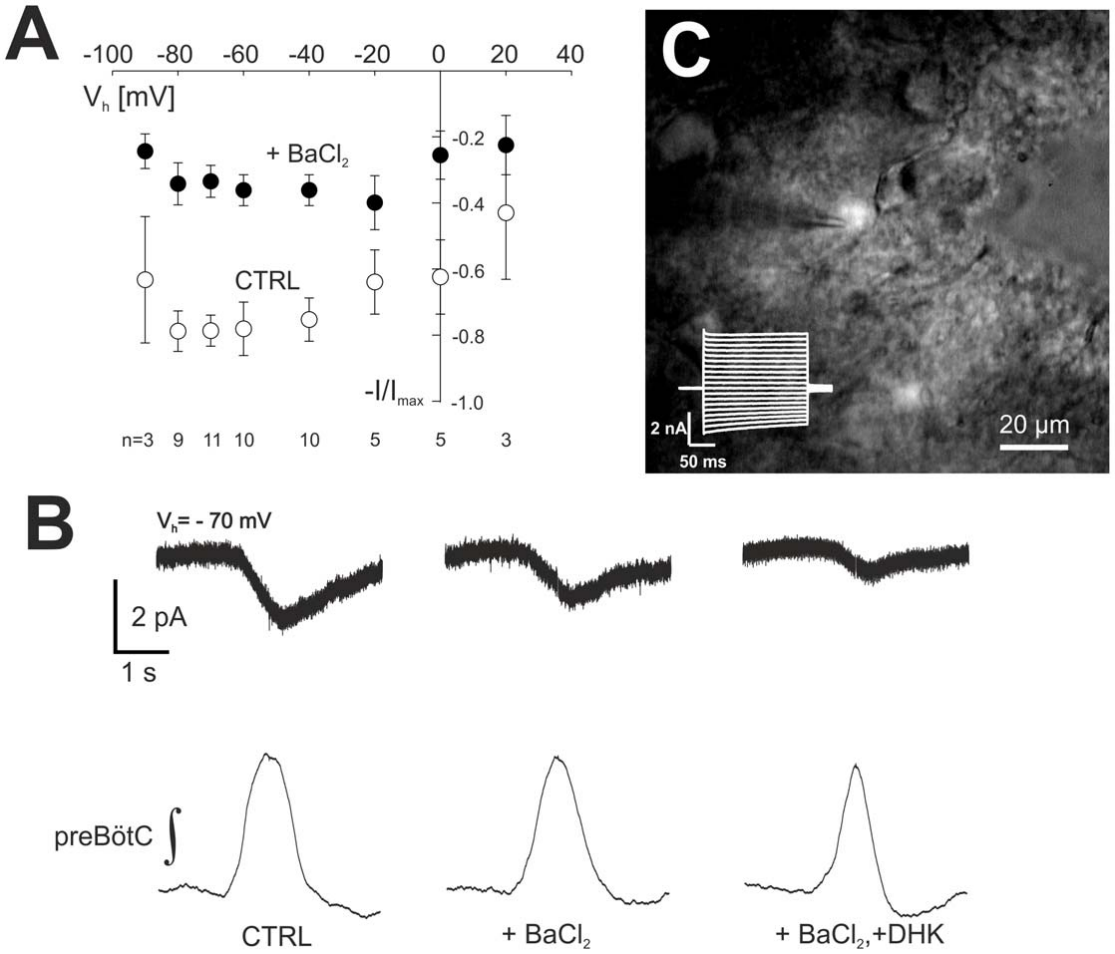
Analysis of respiratory-rhythmic astrocytic currents (I_resp,A_). In panel (**A**) the effect of BaCl_2_ on I_resp,A_ is shown. Data, for each cell normalized (I/I_max_) to the largest current measured over the range of holding potentials, are given for the different holding potentials from −90 mV to +20 mV. Error bars indicate mean ± SEM. The number of cells is indicated below each set of data points. Panel (**B**) shows cycle-averaged currents (holding potential −70 mV) that were recorded from the rhythmic astrocyte (**C**) under control conditions, in the presence of barium (BaCl_2_, 100 µM) and after additional inhibition of glutamate uptake by dihydrokainate (DHK, 300 µM). The cycle-averaged traces of the corresponding integrated preBötC field potential are depicted underneath.

To test for periodic membrane current transients in astrocytes of the preBötC that coincide with rhythmic neuron discharges, we performed whole-cell voltage-clamp recordings from fluorescently labeled astrocytes in the slice preparation. We recorded from a total of 569 fluorescent astrocytes (Figure 1A). As typical, these astrocytes exhibited predominantly passive currents that were distinguished by a linear current-voltage relationship in whole-cell recordings (Figure 1D). Fifty-nine of these astrocytes (10.4%) also exhibited membrane current fluctuations (I_resp,A_) that were in phase with the rhythmic discharges of preBötC neurons. Since I_resp,A_ current amplitude was imbedded to a large extent in background noise (figure 1B), it was not possible to measure current accurately from the raw data. Thus we used cycle triggered averaging to estimate the amplitude, which in 27 astrocytes was –5.9±0.7 pA (mean ± SEM) at V_hold_ = −70 mV (figure 1C). I_resp,A_ was recorded as an inward current at clamping potentials between −90 mV and +20 mV (see figure 2A).

### Rhythmic currents do not involve the activation of additional ion channels

In a next step we tried to determine if I_resp,A_ results in a change of the membrane resistance induced by activation of ion channels during neuronal bursts. PreBötC astrocytes have a low membrane resistance (R_m_) at rest (Graß et al., 2004) constraining the detection of small changes of the membrane conductance during I_resp,A_. Assuming that I_resp,A_ amplitude varies with the degree of neuronal synchronization in the network, we blocked GABAergic transmission with bicuculline (20 µM) to elicit much more intense neuron discharges activity. Under these conditions giant bursts occurred that were accompanied with larger and longer lasting inward currents in the astrocyte (figure 1E). Even under these conditions, I_resp,A_ was not accompanied by changes of input resistance (28.9±15.4 MΩ as compared to the burst intervals 29.0±15.4 MΩ; n = 3). This observation is in line with the assumption that I_resp,A_ reflects fluctuations of the potassium equilibrium potential induced by a rhythmic elevation of the extracellular potassium activity around the astrocyte.

### Barium reduces rhythmic currents in astrocytes

Resting membrane potential in astrocytes of the ventral respiratory column is largely dominated by Kir4.1-channels [14], so we tested whether blockade of K_ir_-channels with Ba^2+^ affects I_resp,A_ amplitude. BaCl_2_ (100 µM) decreased I_resp,A_ amplitude by more than 50%, from −6.7±0.7 pA to −3.0±0.4 pA (V_hold_ = −70 mV; n = 12, p<0.01). Barium induced a reduction of I_resp,A_ at all holding potentials between −90 mV and +20 mV (figure 2A). The resulting parallel shift of the IV-relationship is compatible with the assumption that I_resp,A_ in preBötC astrocytes partially reflects changes of the potassium equilibrium potential. The barium effect did not appear to be linked to changes in the neuronal network activity. Although burst frequency did increase to 0.15±0.02 Hz in the presence of BaCl_2_ (Ctrl: 0.11±0.01 Hz; n = 12; p<0.05), neither the amplitude of neuron field potentials nor its duration at half-maximal amplitude changed (0.79±0.03 s (Ctrl) vs. 0.75±0.04 s with BaCl_2_).

### Glutamate transporter currents contribute to I_resp,A_

We tested whether glutamate released from inspiratory neurons in the preBötC is contributing to I_resp,A_ by measuring the effect of blocking glutamate transporters, which are widely expressed on preBötC astrocytes [14], [22]. In the presence of BaCl_2_, dihydrokainate (DHK, 300 µM), a selective blocker of GLT-1 (EAAT2) further reduced I_resp,A_ (V_hold_ = −70 mV) amplitude by 68% from −3.8±1.2 pA to −1.4±0.7 pA (figure 2B; p<0.05, n = 5). Integrated preBötC burst-amplitude was unchanged by DHK, but half-width-duration of the bursts was reduced from 0.77±0.07 s to 0.56±0.07 s (p<0.05, n = 5) and burst frequency increased, from 0.13±0.02 Hz to 0.20±0.03 Hz (p<0.05, n = 5).

### Metabotropic glutamate receptors elicit calcium signals in preBötC astrocytes

Previous studies from this laboratory demonstrated that glutamate triggers calcium signaling in astrocytes of the ventral respiratory region of the medulla [15], [23], and other studies have shown that group I metabotropic glutamate receptors promote calcium release from intracellular stores and couple neuronal activity to calcium signals in nearby astrocytes of rat cortex, hippocampus and suprachiasmatic nucleus [24], [25].

**Figure 3:**
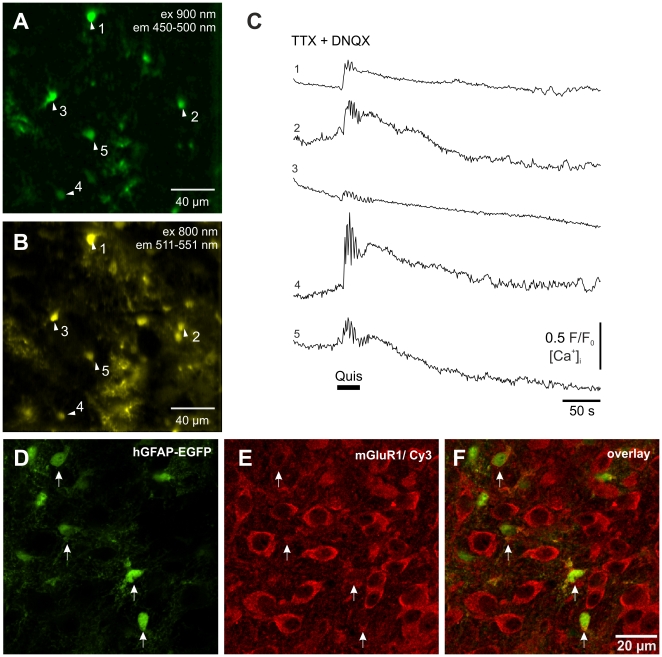
Calcium signals in preBötC astrocytes evoked by activation of mGluR1-receptors. (A–C) Images show (**A**) the distribution of astrocytes identified by 900 nm 2-photon excitation and a CFP emission filter (BP 450–500 nm) and (**B**) Oregon Green BAPTA-1 AM staining **(**800 nm excitation and BP 511–551 nm emission filter). (**C**) Fluorescence traces from astrocyte somata shown in panel (A) in presence of DNQX and TTX. Application of quisqualate (5 µM) evoked a robust calcium elevation in 4 out of 5 astrocytes. (**D–F**) Astrocytic mGluR1-receptor expression is confirmed by immunohistochemistry. Panel (**D**) shows the confocal image of the EGFP-expressing astrocytes (green), and (**E**) the mGluR1-receptor expression. The arrows indicate astrocytes that express mGluR1 receptors (Cy-3, red). Note that neighboring neurons also show a high level of mGluR1-expression. In panel (**F**) the overlay of (**D**) and (**E**) is depicted.

In the present study, we tested whether group I metabotropic receptors contribute to glutamatergic Ca^2+^ signaling in preBötC astrocytes. Two-photon excitation microscopy revealed that the type I agonist quisqualate (5 µM) induced robust astrocyte calcium transients. We co-applied the AMPA/KA receptor blocker DNQX (25 µM) in these tests, since quisqualate has agonistic effects on AMPA receptors as well. In the presence of DNQX and 0.5 µM TTX, quisqualate induced calcium signals in 53±29% of EGFP-labeled preBötC astrocytes in 4 slices. As shown in figure 3, mGluR activation elicited short oscillatory calcium transients that rode on top of a much larger, longer lasting calcium elevation (figure 3C).

Astrocyte group 1 mGluR-expression was also demonstrated by immuno-labeling. We observed mGluR1a-receptors antibody-staining in 63.6±7.1% of the EGFP-labeled astrocytes. Labeling was found on cell bodies as well as on proximal processes (figure 3D–F).

### Rhythmic membrane current fluctuations in preBötC astrocytes are not accompanied by rhythmic Ca^2+^ signals

**Figure 4:**
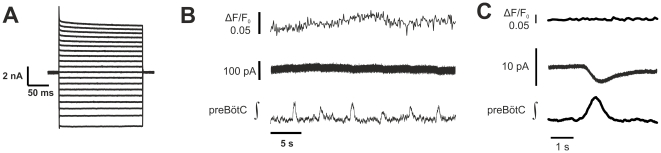
Astrocytes do not exhibit rhythmic calcium signals. (**A**) Current steps evoked in a EGFP-expressing astrocyte by depolarizing and hyperpolarizing voltage steps (10 mV increments) from a holding potential of −70 mV to potentials between −150 to +30 mV. This type of current responses to voltage steps is typical for a passive astrocyte. Panel (**B**) shows calcium signals (ΔF/F_0_) and membrane current (pA) recorded from the particular astrocyte characterized in panel (A), along with simultaneously recorded field potentials (preBötC ∫). In this example, the fluorometric calcium signals (**B, Cc**) were obtained with Calcium orange (200 µM) loaded via the recording pipette. Rhythmic current fluctuations are buried in the noise but are unmasked by cycle triggered averaging in (C). No phase-locked astrocytic calcium signal could be detected.

We recorded from 15 voltage-clamped astrocytes that exhibited rhythmic inward current fluctuations and were dialyzed with calcium indicator dye through the patch pipette. Cycle averaged I_resp,A_ amplitude was −3.17±3.88 pA. Calcium signals synchronized with preBötC field potentials were detected neither in the soma (figure 4) nor in the dendritic compartments of the astrocytes.

**Figure 5:**
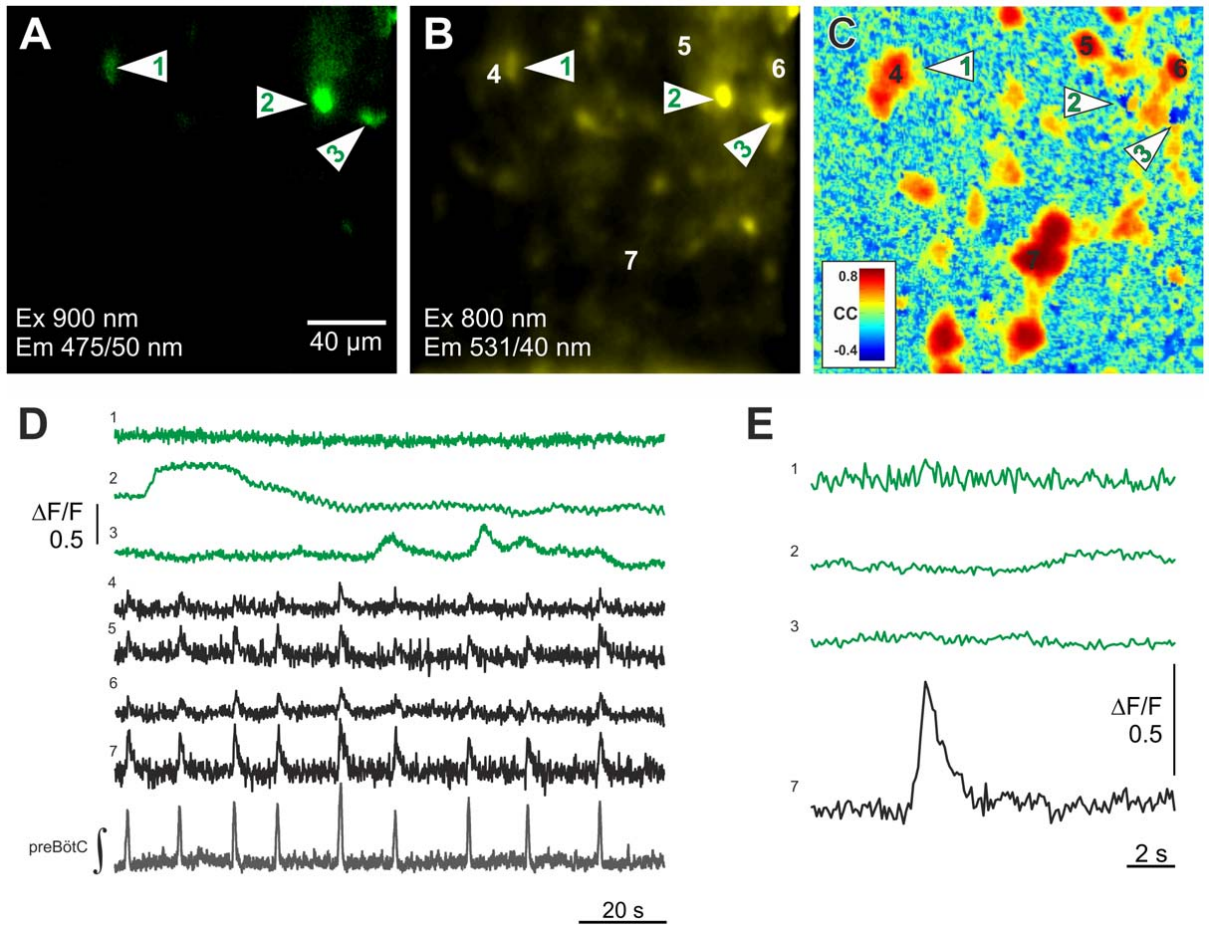
Lack of respiratory-rhythmic calcium signals in astrocytes of the pre-Bötzinger Complex. The figure shows an example of 2-photon calcium imaging from identified astrocytes in the pre-Bötzinger Complex in the presence of bicuculline (20 µM) and strychnine (10 µM). EGFP astrocytes (**A**) were labeled with Oregon Green BAPTA-1 AM (OGB-1 AM, **B**). (**C**) Cross correlation (CC) maps of OGB-1 AM fluorescence were calculated for each image series between each pixel and a respiratory neuron (cell 7). In panel (**D**) the OGB-1 AM fluorescence signals from three astrocytes (1–3) and four respiratory neurons (4–7) are depicted with the integrated network output (preBötC ∫). Astrocytes show spontaneous calcium oscillations that were not phase-locked to the neuronal activity. Additionally, as shown in panel (E) the cycle-averaged data of these recording did not reveal any respiratory-rhythmic calcium signal in the astrocytes.

Two–photon imaging of cells loaded with Oregon Green BAPTA-1 AM (OGB-1 AM) was also carried out to detect Ca^2+^ signals simultaneously in preBötC astrocytes and neurons on the surface and deeper in the slice. Calcium signals were measured in 14 slices from 300 fluorescent-protein labeled astrocytes and from 103 respiratory neurons that were located within 50 µm of the labeled astrocytes (figure 5). Thirty-eight astrocytes (12.7%) exhibited spontaneous fluctuating calcium signals. The calcium signals, however, were not correlated with and entrained by preBötC neuron discharges.

Ongoing neuronal activity in the rhythmic medullary slices might have been too low to produce significant calcium signals in preBötC astrocytes. Therefore we increased the neuronal activity in the network by blockade of inhibition. Bath application of strychnine (10 µM) and bicuculline (10 µM) increased the number of neurons that we could record a rhythmic calcium signal from to 323 (n = 13 slices), twenty-one of them showing “giant” presumably epileptic bursts. After blockade of inhibition, spontaneous calcium fluctuations were evident in 61 (19.9%) of the 307 astrocytes (figure 5D). In two astrocytes, a solitary large calcium signal coincided with neuronal epileptiformic bursts.

Cycle triggered averaging during blockade of synaptic inhibition did not uncover rhythmic calcium signals in 304 of 307 astrocytes (99%; figure 5E). In 3 others, rhythmic Ca^2+^-signals overlapped astrocyte (EGFP) fluorescence. However, the signals might have originated from neuronal processes located in the vicinity of the astrocytes, because cross-correlation activity maps (CC; see methods) only partially overlapped with EGFP-fluorescence. Of greater significance was that the temporal signature of the calcium transients was indistinguishable from a neuronal calcium signals. Taken together, we can conclude from these data that neuronal activity does not trigger respiratory-rhythmic calcium signal in preBötC astrocytes, even under conditions of increased excitation.

### Blockade of glutamate transporters in preBötC astrocytes evokes complex calcium signals and abolishes neuron discharges

**Figure 6:**
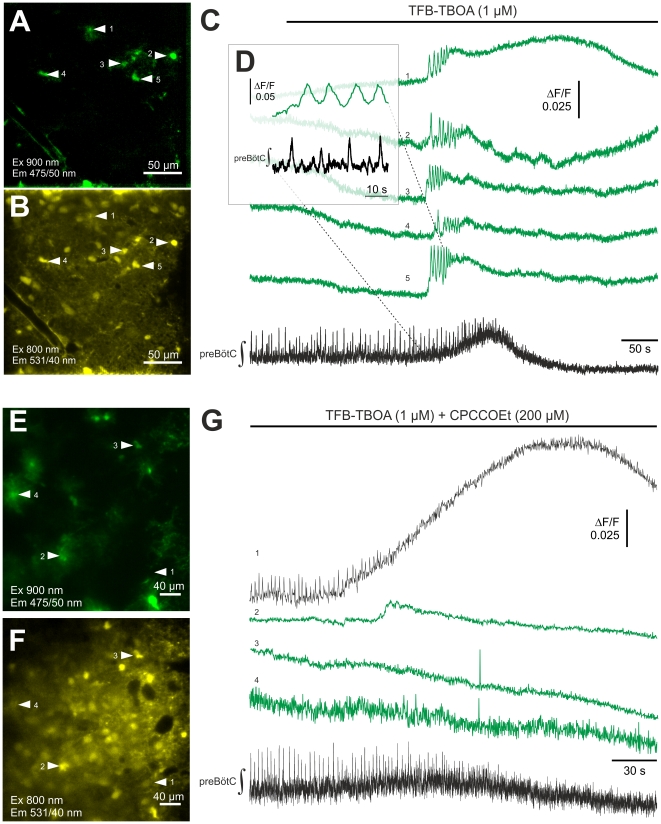
Inhibition of astrocytic glutamate transport elicits robust calcium signals in astrocytes. Panels (**A**–**D**) show an example of fluorometric calcium imaging during glutamate transport: Panel (**A**) identifies astrocytes, which were loaded with the calcium indicator Oregon Green BAPTA-1 AM (panel **B**). (**C**) A blockade of astrocyte glutamate transporters by TFB–TBOA (1 µM) elicited calcium signals in astrocytes (green traces) that were, as shown in (**D**), not phase-locked to preBötC neuronal activity (preBötC ∫). In the second example (**E–G**) the effects of glutamate transport inhibition are investigated after mGluR1-blockade. When the incubation of the mGluR1-antagonist CPCCOEt (200 µM) was started 10 min before the application of TFB-TBOA the astrocytic calcium signals were suppressed. (**G**) Original OGB-1 AM calcium traces are shown from one respiratory neuron (1) and three astrocytes (green traces). Panel (**E**) shows location of the corresponding EGFP-labeled astrocytes and panel (**F**) the distribution of the OGB-1-AM labeling.

We analyzed effects of blocking astrocyte glutamate transporters on Ca^2+^ signaling in astrocytes and neuron discharge properties in preBötC slice preparations. Bath application of TFB-TBOA (1 µM), a potent blocker of both glial glutamate transporters [26], evoked calcium signals in 22 of 25 (88%) astrocytes (n = 5 slices, figure 6). Complex Ca^2+^ waveforms were detected and consisted of relatively short bursts superimposed on larger, prolonged and slow decaying waves (figure 6D). The signal complexes were similar in appearance to those produced by mGluR1 activation.

In all slices, the complex astrocyte Ca^2+^ signals were followed either by an initial intense and sustained discharge of preBötC neurons culminating in discharge abolition (n = 3; see figure 6 D), or by gradually diminishing discharge intensity without antecedent increase until discharge activity was absent (n = 2). The latency to complete blockade of the respiratory activity was 538±115 s (n = 5).

In 5 additional experiments, slices were pretreated for 10 minutes with an mGluR1 receptor antagonist (CPCOOEt, 200 µM) before TFB-TBOA application. In all experiments, TFB-TBOA abolished neuron discharge activity (see figure 6 G), although EGFP-labeled astrocytes did not exhibit oscillatory calcium signals after blockade of mGluR1 receptors and glutamate transporters in 3 slices. In a fourth slice, one astrocyte exhibited a small calcium signal increase (figure 6 E–G). In a fifth, 90% of the EGFP-expressing astrocytes exhibited a calcium signal complex after application of CPCOOEt.

## Discussion

Our general aim in this study was to identify key factors that promote functional coupling between astrocytes and neurons in a critical area of respiratory rhythm control in the brainstem. We analyzed rhythmic membrane currents in astrocytes and investigated functional coupling between preBötC astrocytes and the local neuronal network. With 2-photon calcium imaging, we tested whether neural network discharges trigger Ca^2+^ signals in astrocytes. We measured the effects of glutamate uptake inhibition and mGluR1 activation on astrocyte calcium signaling. Our principal findings were: (1) Rhythmic inwardly directed currents (I_resp,A_) in astrocytes, heretofore not described in other studies, are induced by potassium ion efflux and glutamate release from preBötC inspiratory neurons discharging rhythmically with the respiratory cycle. (2) Non-physiological high levels of glutamate are required for the generation of astrocyte Ca^2+^ waves and therefore coupling between astrocytes and the preBötC respiratory neuronal network, and (3) astrocyte metabotropic glutamate receptors play a role. (4) Glutamate uptake by astrocytes protects preBötC respiratory neurons against excitotoxicity and discharge arrest. Each of the main findings is discussed in the paragraphs that follow.

### Origin and significance of rhythmic membrane currents in preBötC astrocytes

Rhythmic, inwardly directed currents entrained to neuron population discharges were detected in 10% of preBötC astrocytes (figure 1 B,C). We can partially attribute the current fluctuations (I_resp,A_) recorded from astrocytes in the present investigation to periodic elevations in [K^+^]_e_, produced by efflux from discharging neurons. Rhythmic fluctuations in extracellular potassium activity were previously measured in the ventral respiratory column and ranged from 50 µM to 1.5 mM [4], [5]. Such a [K^+^]_e_ change results in shifts of the potassium equilibrium potential at the astrocytes. With preBötC astrocytes having a low membrane resistance [22] one can measure these changes as an inward current in whole-cell voltage-clamp experiments [27]. Previous studies from this laboratory demonstrated that K-currents are large in astrocytes of the ventrolateral respiratory column [22], [23]. The resting membrane potential of preBötC astrocytes is mainly regulated by barium-sensitive Kir4.1 channels [14]. In the present investigation, Ba^2+^ resulted in a robust reduction of I_resp,A_ (figure 2), an observation that is compatible with results from the laboratory, showing a strong reduction of the [K^+^]_e_-induced inward current in preBötC-astrocytes from Kir4.1 knock-out mice [14].

Another component of I_resp,A_ is mediated by electrogenic glutamate uptake via GLT-1. Using the GLT-1 antagonist DHK at a concentration that preserves the network activity intact, we were able to block a significant amount of the astroglial I_resp,A_.

Since astrocyte input resistance remains unchanged even when neuron field discharges are greatly intensified by blocking GABAergic synaptic inhibition (figure 1e), we can conclude that the measured current in astrocytes do not involve currents through ionotropic transmitter receptors.

### Lack of coupling between astrocytes and the local preBötC network during physiological activity

Astrocyte cytosolic calcium oscillations are thought to mediate a bidirectional communication of astrocytes and neurons. Transmitter molecules and modulators released from neurons have been shown to elicit astroglial calcium signals in many parts of the central nervous system [28], [29] including the respiratory network [15], [16]. Through intercellular coupling via gap junctions or by the astroglial release of glutamate, and also ATP or D-serine, the calcium signal can spread to other astrocytes or affect neuronal excitability. The extent of coupling between astrocytes and between astrocytes and neurons can be regenerative and widespread or limited. Furthermore coupling was shown to be bidirectional, with astrocytes influencing neurons and vice versa [29], [30].

In contrast to the results from other brain regions we found no evidence to suggest that calcium dependent signaling directly from astrocytes to the neighboring neuron occurs during physiological network activity in the pre-Bötzinger Complex. Although we observed spontaneous calcium oscillations in astrocytes of the preBötC during constitutive activity, these calcium signals were often restricted to one astrocyte. Even after blockade of synaptic inhibition no global astrocytic calcium signal was detected, thus we conclude that a spread of the calcium waves to neighboring cells is not a common feature of preBötC astrocytes.

In our experiments there was also no detectable functional coupling between individual astrocytes and the neural network in the preBötC. Moreover, preBötC field potentials and single neuron Ca^2+^ transients were rhythmic, but astrocyte Ca^2+^ signals were arrhythmic and asynchronous (figure 5) further illustrating an absence of coupling between astrocytes and neurons. However, it seems that intercellular communication between single astrocytes and the neural network is normally low in the preBötC, perhaps limited by intracellular Ca^2+^ buffering that restricts its spread through astrocyte gap junctions [30]. At the present state we cannot provide a final explanation for this obvious discrepancy and further experiments are necessary to search for the underlying aspects of astroglial heterogeneity.

### Functional relevance of mGluR-1 mediated calcium signaling and extracellular glutamate levels

Astrocytes in the preBötC network are responsive to glutamate [22] and Ca^2+^ transients can be evoked by application of glutamate [15], [23]. In this paper we were able to show that metabotropic glutamate receptors coupled to the PLC/IP3 pathway play a role for glutamate induced astroglial calcium signaling in the preBötC. Since synchronous calcium signaling of astrocytes was only observed after external application of agonists (figure 3) or blockade of glial glutamate uptake (figure 6c) it appears that astrocyte Ca^2+^ signaling is only induced if non-physiological high extracellular glutamate concentrations are reached at the astrocyte. Such conditions occur when the uptake of glutamate released by the local respiratory neurons [31], [32] is blocked pharmacologically (figure 6c) but might also be present during hypoxia when extracellular glutamate levels are significantly increased [33]. In the present study, blockade of mGluR1-receptors prevents glutamate-induced Ca2+ signaling in astrocytes after blockade of glutamate transporter but it does not prevent the respiratory failure, indicating that the astroglial calcium signal, although preceding it, is not causal for the network failure (figure 6 g).

Thus we conclude that the most important role of astrocytes in the preBötC is the control of extracellular levels of neurotransmitters. As figure 6 shows, there are fatal consequences for respiratory neuron viability and rhythm generation when glutamate uptake into astrocytes is impeded.

## Methods

### Breeding of mice

Animals were bred in the animal facility of the University Hospital Göttingen and treated in accordance with the guidelines of the German Physiological Society as well as the regulations of the State of Lower Saxony and the Federal Republic of Germany. The institutional permit number is T19.08. We used transgenic mice, TgN(hGFAP-EGFP) [34] and TgN(hGFAP-mRFP1) [35], expressing green or red fluorescent proteins in astrocytes, respectively. For some initial wide field imaging experiments, NMRI (naval medical research institute) mice were used.

### Slice preparations

Acute brainstem slices of mice from postnatal day 0–11 were prepared as described previously [13]. Briefly, animals were decapitated under diethyl ether anesthesia, the brainstem was isolated and placed in ice-cooled, carbogen-saturated (95% O_2_, 5% CO_2_) artificial cerebrospinal fluid (aCSF: 118 mM NaCl, 3 KCl, 1.5 mM CaCl_2_, 1 mM MgCl_2_, 1 mM NaH_2_PO4, 25 mM NaHCO_3_, and 30 mM D-glucose). The osmolarity was 325–335 mosm/l and the pH 7.4. The isolated brainstem was glued with cyanoacryl glue (Loctite Deutschland GmbH, Munich, Germany) to an agar block. Transverse slices were cut from the caudal medulla at the level of the pre-Bötzinger complex using a vibroslicer (Leica VT 1000S, Leica Instruments, Nussloch, Germany). For experiments, slices were transferred to the recording chamber that was mounted on an upright microscope (Axioscope FS, Zeiss Germany). Slices were kept submerged by a nylon fiber grid [36] and continuously perfused with aCSF at a flow rate of 5–10 ml/min.

### Extracellular recording of preBötC neuron discharges in the brainstem slice preparation

Slices, 600–650 µm thick, were cut from the region of the medulla near the rostral portion of the inferior olive and stored for at least 30 min at room temperature in carbogen-saturated aCSF. After transferring to a recording chamber they were superfused with carbogen-saturated 29–30°C aCSF, while the potassium concentration of aCSF was elevated to 8 mM over a period of 30 minutes to maintain respiratory rhythmic activity. The respiratory rhythm was recorded with extracellular microelectrodes filled with aCSF. Rhythmic population field potentials were amplified (5000–20000 times), band-pass filtered (0.5–2.5 kHz), rectified and integrated using a custom-made amplifier (Electronic workshop, Physiology, Göttingen). Signals were digitized at 10 kHz with interface (ITC-16; InstruTECH/HEKA, Lambrecht) using Axograph software 4 (Axon Instruments, Foster City, CA) or Digidata 1322a interface using pClamp9 software (Molecular Devices, Inc., Sunnyvale, CA), respectively. Digital data were stored on personal computers for later off-line analysis.

### Intracellular whole-cell recordings

Astrocytes were identified by their green fluorescence in the epifluorescence illumination (excitation 488 nm; dichroic mirror 495 nm, Polychrome II, TILL Photonics, Gräfelfing, Germany) as well as by their I-V relationship. Images of astrocytes were acquired with CCD cameras controlled by “Imspector”-software (LaVision BioTec, Bielefeld, Germany) or Imaging Workbench software (Indec Biosystem, Santa Clara, CA, USA).

Whole-cell voltage-clamp recordings from astrocytes were obtained with a Multiclamp 700A amplifier (Axon Instruments, Inc., Forster City, CA, USA). Patch-electrodes were fabricated from borosilicate glass capillaries (Biomedical Instruments, Zülpich, Germany) on a horizontal pipette-puller (Zeitz-Instrumente, Germany) and filled with (in mM) 125 K-Gluconate, 1 CaCl_2_, 2 MgCl_2_, 4 Na_2_ATP, 10 EGTA, 10 HEPES (pH adjusted to 7.2 with KOH). Electrode resistance ranged from 2 to 6 MΩ. Astrocytic membrane currents were sampled at 10 kHz, low-pass filtered at 0.6–1 kHz. The amplifier was controlled by Multiclamp 700A Commander software and Clampex/pClamp 9 (Molecular Devices, Inc., Sunnyvale, CA). For some early experiments we used a L/M-PCA patch clamp amplifier (E.S.F. electronic., Friedland Germany) connected to an interface (ITC-16; Instrutech/HEKA, Lambrecht) that was controlled by “Pulse” software (HEKA, Lambrecht, Germany).

For single-cell calcium measurement of identified astrocytes, electrodes were filled with an intracellular solution containing (in mM): 144 KCl, 0.4 Na-GTP, 2 MgCl_2_, 4 Mg-ATP, 10 HEPES and 100-200 µM Oregon Green BAPTA-1 (n = 5), Fluo 8L (n = 4) and Calcium Orange (n = 6), respectively.

### Cycle triggered averaging of whole-cell currents

To facilitate the detection of small currents in astrocytes we used a Matlab® routine (Mathworks Inc., Natick, MA, USA) that processed membrane current recordings from consecutive respiratory cycles. Peaks of the rhythmic integrated field potential bursts were used to align whole-cell-current for averaging. Whole-cell currents from 5 s before to 15 s after the peak of a respiratory burst were averaged from at least 6 respiratory cycles. Averaged peak currents were analyzed using IGOR Pro (WaveMetrics, Oregon, USA). Mean ± SEM values were calculated with SigmaPlot software (Systat Software GmbH, Erkrath, Germany).

### Cell loading for calcium imaging

Multi-cell bolus loading was performed as described in detail earlier [37]. Briefly, 50 µg Oregon Green BAPTA-1 AM (OGB-1, Molecular Probes, Eugene, OR) was dissolved in DMSO (5 µl) containing 20% Pluronic F-127 (Molecular Probes, Karlsruhe, Germany), and stored at -20°C in 0.5 µl aliquots until used. For injection, one aliquot of this stock solution was dissolved in 5–12 µl of an extracellular solution containing (in mM) 150 NaCl, 2.5 KCl, 10 HEPES (pH adjusted to 7.4). At a final concentration between 0.3 and 0.8 mM a small amount of the OGB-1 AM solution was injected (2 bar; 2 min; 50–100 µm below the slice surface) into the preBötC using a patch pipette [37] followed by an incubation period of 30 min to allow for sufficient dye loading.

### Calcium imaging using multifocal 2-photon excitation microscopy

For 2-photon calcium imaging experiments we used multifocal (16 or 32 foci) excitation. The principle arrangement of our microscope (TriMScope, LaVision BioTec, Bielefeld, Germany) was described earlier [37]. Here we used 40× (0.8 NA) or 20× (1.0 NA) water immersion objectives (Zeiss; Oberkochen, Germany), and CCD-cameras (Ixon 885 or Clara; Andor Technology, Belfast, Northern Ireland, or PCO; Sensicam QE; Kehlheim, Germany).

Calcium signals in hGFAP-EGFP expressing astrocytes were detected and analyzed using OGB-1 AM. OGB-1 fluorescence was detected at 800 nm excitation wavelength through a YFP-Filter (BP 511–551 nm), whereas EGFP-fluorescence was detected through a CFP-filter (475–500 nm) with 900 nm excitation wavelength [37]. Optical filters were obtained from AHF Analysentechnik AG (Tübingen, Germany). Astrocytes expressing mRFP1 were identified through 645/75 nm band pass filters by 2-Photon excitation with 720 nm [38] and analyzed using OGB-1 AM (excitation and emission as above).

### Image processing

For offline analysis, “Imspector”-images were exported to TIFF-format and processed by MATLAB (Mathworks Inc., Natick, MA, USA) or with ImageJ software (http://rsb.info.nih.gov/ij/) using some macros from the Wright Cell Imaging Facility (http://www.uhnres.utoronto.ca/facilities/wcif/download.php). To correlate field potential recordings with optical signals, trigger pulses for each image were recorded simultaneously with the electrical signals using pClamp 9 software and Digidata 1322a (Molecular Devices, Inc., Sunnyvale, CA).

Somatic calcium changes were further analyzed after using the ImageJ macro for background subtraction (“rolling ball” algorithm; radius 30–50 pixel). Fluorescence changes of individual cells were then determined using the “multi measure” regions of interest (ROI)-macro from ImageJ. Average ROI-intensities of individual cell somata were expressed as relative changes (ΔF/F_o_) using a custom made IGOR pro (WaveMetrics, Lake Oswego, OR) macro.

To increase signal to noise ratio of rhythmic signals, the image series from consecutive respiratory cycles were averaged using an algorithm for cycle triggered averaging that was implemented in a MATLAB routine as described earlier [37]: Briefly, a region of interest (ROI) was set on an inspiratory neuron. Peaks of calcium transients associated with the inspiratory activity were detected within the ROI. Using the timing of the inspiratory peaks as the reference, optical signals of frames preceding and following that peak were averaged (5–20 times). Additionally, the cross-correlations (CC) between each pixel and the ROI was calculated (For details see [37]).

### Drugs

Electrolytes for aCSF (see above) were purchased from Sigma-Aldrich (Taufkirchen, Germany) and Merck chemicals (Darmstadt, Germany). Drugs were stored at −20°C as stock solutions in the following concentrations: Barium chloride (BaCl_2_, 100 mM in H_2_O), bicuculline (10 mM in H_2_O) and strychnine (10 mM in H_2_O) were purchased from Sigma-Aldrich, while dihydrokinate (DHK; 100 mM in 1N NaOH), DL-threo-β-Benzyloxyaspartic acid (DL-TBOA; 100 mM in DMSO), (3S)-3-[[3-[[4-(Trifluoromethyl)benzoyl]amino]phenyl]methoxy]-L-aspartic acid (TFB-TBOA; 5 mM in DMSO), 7-(Hydroxyimino)cyclopropa[b]chromen-1a-carboxylate ethyl ester (CPCCOEt; 25 mM in DMSO), (RS)-3,5- Dihydroxyphenylglycine (DHPG, 10 mM in 1N NaoH) tetrodotoxin (TTX, 1 mM in acetic acid), 6,7-Dinitroquinoxaline-2,3-dione (DNQX, 10 mM in DMSO) were ordered from Tocris bioscience (Bristol, UK)) and L-Quisqualic acid (20 mM in H_2_O from Alexis biochemical/Enzo Life Sciences (Lörrach, Germany). For the experiments, stock solutions were diluted in aCSF to reach the final concentrations as indicated in the text. Drugs were applied by changing the superfusion of the slice.

### Immunohistochemistry

Brain tissue of four P5 mice was removed and fixed in 4% paraformaldehyde in 100 mM phosphate buffer. For longer storage tissue was stored at 4°C in PBS, 0.01% sodium azide. Immunohistochemical labeling was performed on free-floating vibratome (Leica VT 1000S, Leica Instruments, Nussloch, Germany) slices at room temperature. Brain tissue slices, 40–50 µm thick, were permeabilized for 2 h in 0.1% Triton X-100 and 10% goat serum in PBS at room temperature and incubated over night at 4°C in PBS with primary anti-mGluR1a antibody (rabbit polyclonal, 1:250, Abcam, Cambridge, UK). Slices were washed 3 times in PBS for 10 min and incubated with Cy3-conjugated secondary antibodies (1∶500 and 1∶1000, Dianova, Hamburg, Germany) for 2 h at room temperature. After washing in PBS, slices were mounted on object slides with Immu-Mount (Shandon, Pittsburgh, PA, USA). Digital images were obtained with confocal laser-scanning microscopy (Zeiss LSM 510Meta, Axiovert 200M, Zeiss, Oberkochen, Germany). Images were stored and processed with the Zeiss LSM software or ImageJ.

## Acknowledgments

We especially thank Anja-Annett Grützner for excellent technical assistance, Diethelm W. Richter for continuous support and Frank Kirchhoff (Universität des Saarlandes) for providing transgenic mice. Yoshitaka Oku (Hyogo College of Medicine) wrote the Matlab®-macros for cycle-trigged-averaging. We owe him special thanks. Peter M. Lalley (University of Wisconsin-Madison) read earlier versions of the manuscript and helped us immensely with critical comments and English editing.
